# Prognostic value of procalcitonin and lipopolysaccharide binding protein in cancer patients with chemotherapy-associated febrile neutropenia presenting to an emergency department

**DOI:** 10.11613/BM.2019.010702

**Published:** 2018-12-15

**Authors:** Luis García de Guadiana-Romualdo, Pablo Cerezuela-Fuentes, Ignacio Español-Morales, Patricia Esteban-Torrella, Enrique Jiménez-Santos, Ana Hernando-Holgado, María Dolores Albaladejo-Otón

**Affiliations:** 1 Biochemistry Department, University Hospital Santa Lucía, Cartagena, Spain; 2 Clinical Oncology Department, University Hospital Santa Lucía, Cartagena, Spain; 3 Hematology Department, University Hospital Santa Lucía, Cartagena, Spain

**Keywords:** febrile neutropenia, prognosis, Multinational Association for Supportive Care in Cancer, procalcitonin, lipopolysaccharide binding protein

## Abstract

**Introduction:**

Cancer patients with chemotherapy-induced febrile neutropenia are a heterogeneous group with a significant risk of serious medical complications. In these patients, the Multinational Association for Supportive Care in Cancer (MASCC) score is the most widely used tool for risk-stratification. The aim of this prospective study was to analyse the value of procalcitonin (PCT) and lipopolysaccharide binding protein (LBP) to predict serious complications and bacteraemia in cancer patients with febrile neutropenia, compared with MASCC score.

**Materials and methods:**

Data were collected from 111 episodes of febrile neutropenia admitted consecutively to the emergency department. In all of them, MASCC score was calculated and serum samples were collected for measurement of PCT and LBP by well-established methods. The main and secondary outcomes were the development of serious complications and bacteraemia, respectively.

**Results:**

A serious complication occurred in 20 (18%) episodes and in 16 (14%) bacteraemia was detected. Areas under the receiver operating characteristic curve (ROC AUC) of MASCC score, PCT and LBP to select low-risk patients were 0.83 (95% confidence interval (CI): 0.74 - 0.89), 0.85 (95% CI: 0.77 - 0.91) and 0.70 (95% CI: 0.61 - 0.78), respectively. For bacteraemia, MASCC score, PCT and LBP showed ROC AUCs of 0.74 (95% CI: 0.64 - 0.82), 0.86 (95% CI: 0.78 - 0.92) and 0.76 (95% CI: 0.67 - 0.83), respectively.

**Conclusion:**

A single measurement of PCT performs similarly as MASCC score to predict serious medical complications in cancer patients with febrile neutropenia and can be a useful tool for risk stratification. Besides, low PCT concentrations can be used to rule-out the presence of bacteraemia.

## Introduction

Febrile neutropenia is a well-known complication induced by chemotherapy and one of the most frequent oncologic emergencies encountered by physicians in an emergency department (*1*). As this condition predisposes to infection and related serious complications, it requires establishing immediately the prognosis and treatment of the patient. Besides, because oral or ambulatory treatment is a safe alternative to inpatient management, the identification of patients being at low risk for complications, candidates for outpatient management, is essential to avoid the overtreatment and unnecessary hospital admissions (*1*).

The most widely used model to identify low-risk patients with febrile neutropenia is Multinational Association for Supportive Care in Cancer (MASCC) risk index score, based on seven independent predictive factors that can be assessed at fever onset, without laboratory results (*2*). However, it has also limitations: some of its components are objective (*e.g*., patient age), but two components are inherently subjective: burden of disease and presence of dehydration requiring *iv.* fluids. Besides, its ability to predict serious complications is not optimal, because they occur in up to 9% to 15% of episodes classified as low-risk (*3*). Although the use of MASCC risk score has been recommended in the most recent international guidelines, Baugh *et al.* have recently concluded that guideline concordance was low among low-risk patients, with management, including admission to hospital and parenteral antibiotic regimens, tending to be more aggressive than recommended (*4*, *5*).

Since the infectious aetiology is the main cause of complications in cancer patients with febrile neutropenia, several studies have evaluated the role of infection biomarkers, such as procalcitonin (PCT), to improve the performance of MASCC score for risk-stratification or have included it in new prognostic models (*6*, *7*). Less known is the prognostic role of another infection biomarker, lipopolysaccharide binding protein (LBP), which has a diagnostic accuracy for infection similar to PCT, recently reported by our group in a previous cohort including febrile neutropenia episodes presenting to the emergency department of our hospital from 2010 to 2012 (*8*).

The purpose of this prospective diagnostic accuracy study was to evaluate the value of PCT and LBP, measured in the first blood collected at the emergency department, as predictors of serious complications and bacteraemia during an episode of febrile neutropenia in cancer patients, compared with MASCC score.

## Materials and methods

### Study design and subjects

From November 2012 to June 2014, a single-center prospective observational cohort study was performed at the emergency department (ED) of the University Hospital Santa Lucía (Cartagena, Spain). Inclusion criteria were adult (≥ 18 years) outpatients with febrile neutropenia who received chemotherapy for underlying malignancy within 5 weeks prior to admission to the emergency department. Patients previously treated at other hospitals for neutropenic episodes and then transferred to our hospital were excluded. Neutropenia was defined as a neutrophil count of < 0.5 x10^9^/L, or < 1.0 x10^9^/L with a predicted decrease to < 0.5 x10^9^/L. Fever was defined as a single oral temperature of ≥ 38.3 °C or ≥ 38 °C for ≥ 1 hour (*9*). In our hospital, cancer patients with fever are initially evaluated by emergency physicians. After confirming the presence of neutropenia, an expert clinician in Hematology (for haematological malignancies) or Oncology/Internal Medicine (for solid tumours) re-evaluated the patients and MASCC score was calculated and recorded in the patient´s clinical history.

The study was performed according to the Declaration of Helsinki and approved by the Ethics Committee for Clinical Research at our hospital (TI11/14). Written informed consent was obtained from all patients.

Demographic and clinical data, such as type of cancer, focus of infection, fever duration, MASCC score on admission to emergency department and hemodynamic and biochemical and coagulation tests suggestive of organ dysfunction, were collected from the medical records.

### Outcomes

The main outcome was the development of serious complications after the initial screening and until febrile neutropenia resolution, with modifications of the initial antibiotic therapy allowed. The serious complication outcome was defined as the presence of at least one of the following: hypotension (systolic blood pressure less than 90 mm Hg or need for pressor support to maintain blood pressure), respiratory failure (arterial oxygen pressure less than 60 mm Hg while breathing room air or need for mechanical ventilation), intensive care unit admission, disseminated intravascular coagulation, confusion or altered mental status, congestive cardiac failure seen on chest x-ray and requiring treatment, severe bleeding requiring transfusion, electrocardiogram changes or arrhythmia requiring treatment, renal failure requiring investigation and/or treatment with intravenous fluids, dialysis or any other intervention and any other complication judged serious and clinically significant by the investigator (*2*). Mild symptoms, such as mild pain, nausea, chills, myalgia or arthralgia, were not included in the defined criteria for serious complication. Besides, we defined bacteraemia as a secondary outcome. Although bacteraemia cannot be considered as a complication itself, it is a relatively common condition in cancer patients with febrile neutropenia and the occurrence of complications and mortality is higher in bacteremic episodes; therefore, early prediction of bacteraemia in these patients might be important to tailor empirical treatment to cover the increased risk (*10*). Bacteraemia was defined as the presence of alive bacteria in blood in one or both blood culture sets. In cases where only one set of microorganisms usually considered as contaminant, such as coagulase-negative staphylococci, viridans streptococci, *Propionobacterium acnes*, *Corynebacterium sp*. and *Bacillus sp*. was detected, the isolation was considered as contamination and therefore not defined as bacteraemia (*11*).

In patients admitted to ward or intensive care unit from ED, both outcomes were determined by only two clinicians based on the complete patient’s records, without knowing biomarker concentrations. For discharged patients from ED, at the discretion of the treating physicians, notes from subsequent visits up to 30 days post-discharge were reviewed to evaluate for adverse outcomes.

### Blood sampling

Blood samples were collected by venipuncture into tubes with separator gel (BD Vacutainer SST II Advance 8.5 mL) on presentation to emergency department. Samples for PCT and LBP were centrifuged at 3000 rpm for 10 minutes. Separated serum was used for the immediate measurement of PCT and remaining serum was frozen and stored at - 80 °C until LBP measurement was carried out, according to the manufacturer´s recommendations about LBP stability.

For microbiological investigations, collection of biological samples was ordered by physicians in the ED. According to the procedures of ED in our hospital, blood culture was required in all the episodes. Blood for culture was collected in two sets of bottles (BACTEC plus Aerobic/F, Becton Dickinson Microbiology Systems, Cokeysville, USA), one aerobic and one anaerobic, prior to initiation of antibiotic therapy, and incubated for at least 5 days (BD BACTEC FXâ system, Becton Dickinson Microbiology Systems, Cokeysville, USA). All isolates were identificated following standard procedures (Microscanâ WalkAway system, Siemens Healthcare Diagnostics, Los Angeles, USA). Besides, cultures from other origins, such as urine, respiratory tract, cerebrospinal fluid, stool, or wound exudates, were collected according to the clinical judgement of treating clinicians, according to the standard procedures of Microbiology and Parasitology Department.

## Methods

Serum PCT concentrations were measured on a Cobas e411 analyser (Roche Diagnostic, Mannheim, Germany) by an electrochemiluminiscence assay. According to manufacturer´s data, detection limit, functional sensitivity and measurement range were 0.02 mg/L, 0.06 mg/L and 0.02 to 100 mg/L, respectively, and the total imprecision, expressed as coefficient of variation (CV), ranged from 0.9% for a mean level of 10.2 mg/L to 1.3% for a mean level of 0.52 mg/L.

Concentrations of LBP were measured with a chemiluminescent assay on an Immulite 2000 analyser (Siemens Healthcare Diagnostics, Los Angeles, USA). According to the manufacturer´s literature, detection limit, functional sensitivity and measurement range were 1.2 mg/L, 1.5 mg/L and 1.2 to 170 mg/L, respectively, and the total CV ranged from 4.1% for a mean level of 107 mg/L to 6.0% for a mean level of 5.31 mg/L.

### Statistical analysis

The Kolmogorov-Smirnov and Shapiro-Wilks tests were used to assess the normality of distribution of investigated parameters. All continuous variables in our study were distributed non-normally. They were presented as medians (interquartile range, IQR) and compared with Mann-Whitney test. Categorical variables were expressed as frequencies and percentages and compared with Chi-square test. To evaluate the discrimination ability of the tested variables, area under the receiver operating characteristic curve (ROC AUC) were calculated and differences among ROC AUCs were assessed with the Hanley and Mc Neil test. We additionally estimated the optimal ROC curve-derived cut-offs by the Youden index. A binary logistic regression analysis was performed to identify predictors for serious complications and bacteraemia, including both MASCC score and biochemical markers. The values P < 0.05 were considered statistically significant. Software packages SPSS version 20 (SPSS Inc., Chicago, USA) and MedCalc v.15.0 (MedCalc Software, Ostend, Belgium) were used for statistical analyses.

## Results

During the study period, 114 consecutive episodes of chemotherapy associated febrile neutropenia were documented in 105 patients. Three episodes in 3 patients were excluded due to the lack of blood sample for biomarker measurements. Finally, the population study included 111 episodes of febrile neutropenia in 102 patients, with a median age of 63 years (range: 21 - 85), and 40 (39%) men and 62 women (61%). From that number, 81 had a solid tumour (80%), including breast tumour (N = 36; 44%), lung tumour (N = 22; 27%), gynaecologic tumours (N = 8; 10%), urologic tumours (N = 9; 11%), digestive tumours (N = 4; 5%) and other tumours (N = 2; 3%); and 21 (20%) had haematological malignancies, including Hodgkin and non-Hodgkin lymphoma (N = 16; 76%) and acute leukaemia and myelodysplastic syndromes (N = 5; 24%). Characteristics of febrile neutropenia episodes (N = 111) are listed in Table 1. Most of the episodes, 87 (78%), were classified as having a low risk of complication (MASCC risk index score ≥ 21). Infection was documented in 57 episodes (51%). The most common sources of infection were urinary (N = 20; 35%) and respiratory tracts (N = 15; 26%). In patients in whom infection was clinically or microbiologically documented no differences on biomarker levels were found according to the focus of infection. Serious complications were observed in 20 episodes (18%), including 10 deaths (9%), and 16 (14%) had positive blood cultures, 14 (88%) by gram-negative bacteria. The occurrence of serious complications was higher in bacteraemia episodes (43.8% *vs*. 13.7%; P = 0.004).

**Table 1:** Baseline characteristics of febrile neutropenia episodes

**Characteristics**	**Serious complications**			
	**Total** **N = 111**	**No** **N = 91**	**Yes** **N = 20**	**P**
Age (years)	63 (21 - 85)	62 (28 - 85)	70 (21 - 83)	0.105
Male gender, N (%)	42 (38)	28 (31)	14 (70)	0.001
Underlying malignancies, N (%) Solid tumour Haematological malignancy	85 (77) 26 (23)	68 (75) 23 (25)	17 (85) 3 (15)	0.326
MASCC score	24 (21 - 24)	24 (22 - 24)	19 (12 - 22)	< 0.001
Low-risk (MASCC index risk score ≥ 21), N (%) High-risk (MASCC index risk score < 21), N (%)	87 (78) 24 (22)	80 (88) 11 (12)	7 (35) 13 (65)	< 0.001
Fever duration before admission < 24 h, N (%)	74 (71)*	63 (69)	11 (55)	0.411
Granulocyte-stimulating factors, N (%)	99 (89)	81 (89)	18 (90)	0.897
Age is presented as median (range). MASCC - Multinational Association for Supportive Care in Cancer. MASCC score is presented as median (interquartile range). Continuous variables were compared by using Mann-Whitney test. Categorical variables were compared by using Chi-square test. *Data available in 105 febrile neutropenia episodes. Fever duration before admission refers to the time from the onset of fever to admission to the emergency department. Granulocyte-stimulating factors refers to the onset of therapy with granulocyte-stimulating factors when febrile neutropenia was detected. P < 0.05 was considered statistically significant.				

**Figure 1:**
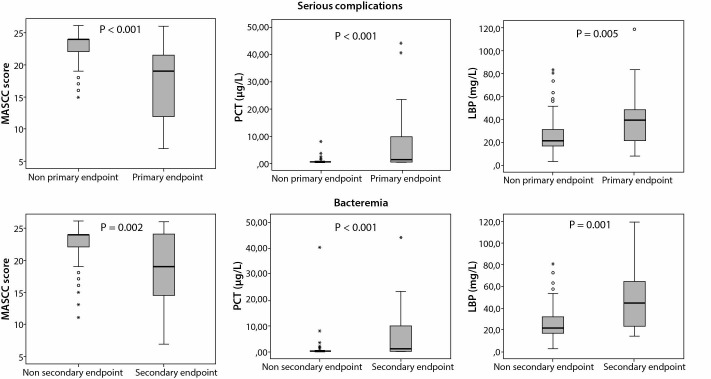
Median (boxplots) values of MASCC score, PCT and LBP in patients developing serious complications (up) and bacteraemia (down) outcomes. In boxplots, the dark line inside the box is the median of the distribution. The bottom and the top of the boxes represent the 1^st^ and the 3^rd^ quartiles and the whiskers represent the intervals containing those values whose distance up to the 1^st^ quartile and from the 3^rd^ quartile is smaller or equal to 1.5 times the interquartile range (IQR). MASCC - Multinational Association for Supportive Care in Cancer. PCT – procalcitonin. LBP - lipopolysaccharide binding protein.

Febrile neutropenia episodes developing serious complications only differed in gender, MASCC score and biomarker levels (Tables 1 and 2, Figure 1). Concentrations of PCT and LBP were also significantly higher in the group of bacteraemia episodes (Figure 1, Table 2). MASCC score was significantly lower in episodes with bacteraemia (Figure 1, Table 2).

**Table 2:** Comparison of MASCC risk index score and biomarker concentration

	**MASCC score**	**PCT (mg/L)**	**LBP (mg/L)**
Non serious complications	24 (22 - 24)	0.13 (0.08 - 0.30)	21.4 (16.2 - 31.4)
Serious complications	19 (12 - 22)	1.02 (0.28 - 11.41)	39.1 (20.8 - 48.9)
P	< 0.001	< 0.001	0.005
Non bacteraemia	22 (22 - 24)	0.13 (0.09 - 0.32)	21.4 (16.2 - 33.2)
Bacteraemia	19 (13 - 24)	0.91 (0.40 - 11.41)	44.2 (22.3 - 69.5)
P	0.002	< 0.001	0.001
Data are expressed as median (interquartile range). Continuous variables were compared by using Mann-Whitney test. MASCC - Multinational Association for Supportive Care in Cancer. PCT – procalcitonin. LBP - lipopolysaccharide binding protein. P < 0.05 was considered statistically significant.			

**Figure 2:**
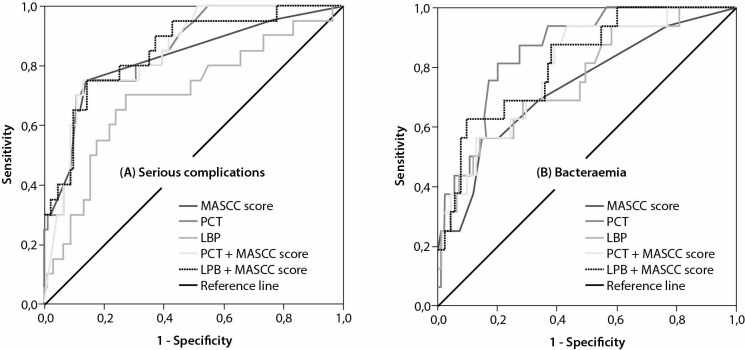
Area under the curve of receiver operating characteristic (with the 95% confidence interval (CI) for the prediction of serious complications (A) and bacteraemia (B). MASCC - Multinational Association for Supportive Care in Cancer. PCT – procalcitonin. LBP - lipopolysaccharide binding protein.

Regarding to the performance of MASCC score and biomarker levels as predictor tools (Figure 2), MASCC score and PCT concentrations, measured at presentation to the ED, showed a similar and good ability to predict the development of serious complications, with ROC AUCs of 0.83 (95% confidence interval (CI): 0.74 - 0.89) and 0.85 (95% CI: 0.77 - 0.91), respectively, both higher than LBP (ROC AUC: 0.70 (95% CI: 0.61 - 0.78), although the difference was marginally significant (P = 0.054) when ROC AUCs of MASCC score and LBP were compared (Table 3). The combination of MASCC score and PCT did not improve the performance of both variables alone. Analysing the episodes that finally developed a serious complication, 7 and 5 episodes were classified as low-risk according to MASCC score (≥ 21) and PCT concentration (< 0.43 µg/L), respectively. Four episodes developing a serious complication showed simultaneously a PCT < 0.43 µg/L and MASCC score ≥ 21.

**Table 3:** Area under the receiver operating characteristic curve of MASCC score and biomarkers to predict serious complications

	**ROC AUC (95% CI)**	**P**	**P-value of comparison *vs*. MASCC score**	**P-value of comparison *vs*. PCT**
MASCC	0.83 (0.74 - 0.89)	< 0.001	-	0.573
PCT	0.85 (0.77 - 0.91)	< 0.001	0.573	-
LBP	0.70 (0.61 - 0.78)	0.005	0.054	0.020
MASCC + PCT	0.84 (0.76 - 0.90)	< 0.001	0.526	0.789
MASCC + LBP	0.85 (0.77 - 0.91)	< 0.001	0.237	0.972
ROC AUC - Area under the receiver operating characteristic curve. CI - confidence interval. MASCC - Multinational Association for Supportive Care in Cancer. PCT – procalcitonin. LBP - lipopolysaccharide binding protein (LBP). Hanley and Mc Neil test was used for comparison of ROC AUCs. P < 0.05 was considered statistically significant.				

For bacteraemia, ROC AUC corresponding to PCT (0.86, 95% CI: 0.78 - 0.92) was higher than those of MASCC score (0.74, 95% CI: 0.64 - 0.82) and LBP (0.76, 95% CI: 0.67 - 0.83), although when both biomarkers were compared the difference was not significant (P = 0.144) (Figure 2, Table 4). Although the combination of MASCC score and PCT increased significantly, the performance of MASCC score alone (0.80, 95% CI: 0.71 - 0.87 *vs.* 0.74, 95% CI: 0.64 - 0.82; P = 0.010), it did not improve the performance of PCT alone (Table 4). The selected cut-offs for MASCC, PCT and LBP and their accuracy to predict the development of serious complications and bacteraemia are listed in Table 5.

**Table 4:** Area under the receiver operating characteristic curve of MASCC score and biomarkers to predict bacteraemia

	**ROC AUC (95% CI)**	**P**	**P - value of comparison *vs.* MASCC score**	**P - value of comparison *vs.* PCT**
MASCC	0.74 (0.64 - 0.82)	0.002	-	0.030
PCT	0.86 (0.78 - 0.92)	< 0.001	0.030	-
LBP	0.76 (0.67 - 0.83)	< 0.001	0.792	0.144
MASCC + PCT	0.80 (0.71 - 0.87)	< 0.001	0.010	0.207
MASCC + LBP	0.82 (0.74 - 0.89)	< 0.001	0.081	0.428
ROC AUC - Area under the receiver operating characteristic curve. CI - confidence interval. MASCC - Multinational Association for Supportive Care in Cancer. PCT – procalcitonin. LBP - lipopolysaccharide binding protein (LBP). Hanley and Mc Neil test was used for comparison of ROC AUCs. P < 0.05 was considered statistically significant.				

**Table 5:** Performance of MASCC risk index score and biomarker levels to predict serious complications and bacteraemia

	**Cut-off**	**Sensitivity (%)**	**Specificity (%)**	**PPV (%)**	**NPV (%)**
**Serious complications**					
MASCC score	< 21	75 (51 - 91)	86 (77 - 92)	53 (34 - 73)	94 (86 - 98)
PCT (mg/L)	≥ 0.43	75 (51 - 91)	86 (77 - 92)	54 (34 - 73)	94 (86 - 98)
LBP (mg/L)	≥ 28.8	70 (46 - 88)	73 (62 - 81)	36 (21 - 53)	92 (83 - 97)
**Bacteraemia**					
MASCC score	< 21	56 (30 - 80)	80 (71 - 88)	32 (16 - 53)	92 (83 - 97)
PCT (mg/L)	≥ 0.34	81 (54 - 96)	80 (71 - 88)	41 (24 - 60)	96 (89 - 99)
LBP (mg/L)	≥ 40.7	56 (30 - 80)	86 (78 - 93)	41 (20 - 64)	92 (84 - 97)
The cut-offs for PCT and LBP were chosen according to Youden index. 95% confidence intervals are presented in brackets. PPV - positive predictive value. NPV - negative predictive value. MASCC - Multinational Association for Supportive Care in Cancer. PCT – procalcitonin. LBP - lipopolysaccharide-binding protein.					

The unadjusted odd ratio (OR) for the development of serious complications in episodes with MASCC < 21, PCT ≥ 0.43 mg/L and LBP ≥ 28.8 mg/L are listed in Table 6. After adjusting for male gender and haematological malignancy, PCT ≥ 0.43 mg/L showed the strongest independent association with the risk of development of serious complications (OR: 10.4, 95% CI: 2.6 - 42.6; P = 0.001) (Table 6). For bacteraemia, in multivariate analysis, PCT ≥ 0.34 mg/L was the only independent predictor of this outcome (OR: 10.1, 95% CI: 1.9 - 54.8; P = 0.008) (Table 6).

**Table 6:** Univariate and multivariate regression to predict serious complications and bacteraemia

	**Univariate**		**Multivariate**	
	**OR (95% CI)**	**P**	**OR (95% CI)**	**P**
	**Serious complications**			
MASCC score < 21	13.5 (4.4 - 41.2)	< 0.001	4.8 (1.2 - 19.0)	0.027
PCT ≥ 0.43 mg/L	18.0 (5.6 - 58.0)	< 0.001	10.4 (2.6 - 42.6)	0.001
LBP ≥ 28.8 mg/L	6.2 (2.1 - 17.1)	0.001	1.2 (0.3 - 5.5)	0.774
	**Bacteraemia**			
MASCC score < 21	6.9 (2.2 - 21.3)	0.001	1.7 (0.4 - 7.6)	0.506
PCT ≥ 0.34 mg/L	17.3 (4.5 - 67.0)	< 0.001	10.1 (1.9 - 54.8)	0.008
LBP ≥ 40.7 mg/L	8.1 (2.6 - 25.6)	< 0.001	1.6 (0.3 - 7.7)	0.574
OR - odd ratio. CI - confidence interval. MASCC - Multinational Association for Supportive Care in Cancer. PCT – procalcitonin. LBP - lipopolysaccharide binding protein. P < 0.05 was considered statistically significant.				

## Discussion

Febrile neutropenia is one of the most common oncological emergencies and the availability of objective criteria for an early risk identification is a critical task for the management of this condition (*1*, *4*). This study has investigated the role of PCT and LBP, compared to MASCC score, as markers for prognosis in adult cancer patients with chemotherapy-associated febrile neutropenia presenting at an ED. The main findings of the present study are: (a) PCT and MASCC score performs similarly to predict serious complications in cancer patients with a febrile neutropenia episode; and (b) for bacteraemia, PCT was the variable with a highest predictive value.

Regarding to the first finding, PCT and MASCC score presented a similar performance to predict serious complications, with ROC AUCs of 0.85 and 0.83, respectively, and higher than that of the other tested biomarker, LBP (ROC AUC: 0.70), with a difference between MASCC score and LBP nearly to significance (P = 0.054). Besides, PCT, measured on admission to the ED, was a strong predictor for serious complications, with an OR of 10.4 for concentrations ≥ 0.43 mg/L.

For MASCC score, our results are in accordance with results of previous studies validating this risk index score. In a recent multicenter study performed in three different emergency departments, Ahn *et al.* reported a ROC AUC of 0.77 for MASCC score to identify low-risk neutropenic fever patients, with a negative predictive value (NPV) of 87.5% to rule-out serious complications, similar to that reported by Uys *et al.* (NPV: 86.4%), slightly lower values than that achieved in our study (94%) (*12*, *13*). A similar performance was recently reported by Ahn *et al.* using a new model including demographic, clinical variables and PCT, achieving ROC AUCs of 0.84 in derivation set and 0.77 in the validation set (*7*).

Previous studies have analysed the prognostic role of biomarkers in febrile neutropenia patients (*13*-*15*). However, the comparison of results reported in these studies is limited due to differences in the definition of outcomes, having been included in some studies events such as bacteraemia, severe sepsis and septic shock or treatment failure as adverse events. In studies using the same adverse events as Klastersky *et al.* to define serious complications, the results reported about the role of PCT are controversial (*2*). Recently, in a haematological population, Michel *et al.* reported a ROC AUC for PCT of 0.92, higher than other biological markers, such as IL-8 (ROC AUC: 0.81) and C-reactive protein (ROC AUC: 0.72), with an optimal cut-off 0.5 mg/L, similar to that chosen in our study (0.43 mg/L), and with a sensitivity and specificity of 80% and 76%, respectively (*15*). Interestingly, in Ahn *et al.* study, similarly to our results, PCT was an independent predictor for complications, with an OR of 5.76 (7). However, in Uys *et al.* study, none of the laboratory parameters, including PCT, was a significant predictor of resolution with or without complications and death (*13*).

To our knowledge, no study has analysed the prognostic value of LBP in febrile neutropenia episodes. We have found that LBP had a prognostic value to predict serious complications significantly lower than that showed by the other tested biomarker, PCT. In other settings, LBP on admission failed to predict mortality in critically ill patients, with ROC AUC < 0.60, although in Villar *et al.* study, serial LBP serum measurements were associated with disease severity and outcomes (*16*, *17*).

In our study, we also evaluated the capacity of MASCC score and tested biomarkers to predict bacteraemia. Similarly to previous studies, the occurrence of serious complications was higher in bacteremic episodes (*10*). Procalcitonin was the variable which presented the highest ROC AUC (0.86) to predict bacteraemia, higher than MASCC score (ROC AUC: 0.74) and with a trend to be superior in comparison to LBP (ROC AUC: 0.76), probably due to the small size of study population and number of episodes of bacteraemia detected. Besides, PCT was the only independent predictor for bacteraemia, with an OR of 10.1 for concentrations ≥ 0.34 mg/L.

The role of MASCC score for prediction of bacteraemia has been recently evaluated by Ahn *et al.*, who reported a ROC AUC slightly higher (0.82) than that found in our study (0.74) (*6*). Similar results have been recently reported by the same group, achieving ROC AUCs of 0.802 and 0.814 in derivation and validation sets, respectively (*7*). In the same study, a model including demographic, clinical and biochemical variables (PCT), achieved ROC AUCs of 0.86 in derivation set and of 0.87 in the validation set, respectively (*7*).

The role of biomarkers to predict bacteraemia and other bloodstream infections has been widely studied in different settings, including patients presenting at ED and adult and child cancer patients with and without neutropenia (*6*, *15*, *18*-*23*). In a recent meta-analysis, Hoeboer *et al.* reported a ROC AUC of 0.78 to predict bacteraemia in patients with suspected infection presenting at an emergency department, decreasing to 0.71 in immunocompromised/neutropenic patients (*24*). In studies carried out in emergency departments including only adult cancer patients with febrile neutropenia, ROC AUCs of PCT ranged from 0.75 to 0.82, values slightly lower than that found in our study (ROC AUC: 0.86), and similar to those reported in a general population presenting at an emergency department, with sensitivities and specificities ranging from 60.5% to 71% and from 82% to 82.3%, respectively (*6*, *18*-*20*, *23*). The optimal cut-off in both studies was 0.5 mg/L, higher than that calculated in ours, 0.34 mg/L, with a sensitivity of 81% and specificity of 80%. Some studies performed in general population at emergency departments recommend the use of a PCT concentration of 0.1 mg/L to rule-out bacteraemia, with NPV ranging from 96.3% to 99.6% (*18*-*20*). Using this cut-off in our study, PCT achieved a NPV of 100%. Similarly to previous studies, PCT was a strong independent predictor for bacteraemia in our study (*6*, *23*).

Fewer studies have evaluated the association between LBP levels and bacteraemia. Therefore, in Ratzinger *et al.* study, conducted in in-patients at standard care wards with suspected infection and systemic inflammatory response syndrome (SIRS), LBP showed a moderate ability to predict bacteraemia, with a ROC AUC of 0.63, similar to the ROC AUC reported by Gille-Johnson *et al.* to predict bacteraemia in an emergency department (ROC AUC: 0.65), but lower than that reported by Gaïni *et al.* in hospitalized patients with suspected infection, with a ROC AUC of 0.74 (*25*-*27*). The results recently reported by Ratzinger *et al.* in adult in-patients with suspected sepsis and SIRS are of interest, because they have reported that the prognostic capacity of LBP for bacteraemia was significantly lower in non-neutropenic SIRS patients (ROC AUC: 0.61) than in neutropenic SIRS patients (ROC AUC: 0.86), also higher than that calculated in our study (ROC AUC: 0.76) (*28*). However, the mechanism explaining these higher LBP levels in neutropenic patients has not been clearly elucidated. Only one prospective study has evaluated the predictive capacity of LBP for bacteraemia/clinical sepsis in the febrile neutropenia setting, but in a paediatric population, achieving a moderate performance, with a ROC AUC of 0.65 (*29*).

We are aware of the limitations of our study. Firstly, this is a single-center study, which could limit the generalization of the results. Moreover, the number of episodes included was not high, which could imply a type-II error when making comparisons and the small number of detected outcomes does not allow robust conclusions to be drawn. Therefore, although recent studies have demonstrated the influence of type of bacteria and infection foci on biomarker levels, we were not able to describe these differences in our population (*30*). However, our study population, although small, is a heterogeneous group, including patients with solid tumours and haematological cancers similarly distributed as compared with other studies and with an incidence of serious complications similar to that reported in previous studies (*7*).

In conclusion, our results suggest that the measurement of an easily measurable and with a short turn-around-time biomarker, PCT, is a useful tool for risk-stratification of cancer patients with febrile neutropenia, with a similar prognostic capacity as MASCC score, and higher than that found for the other tested biomarker, LBP. Besides, in these patients, PCT is a useful marker to rule-out bacteraemia. Further larger studies about the safety and efficacy of using PCT as a single prognostic tool or in combination with risk index scores are required.
